# Brain hemodynamic activity during viewing and re-viewing of comedy movies explained by experienced humor

**DOI:** 10.1038/srep27741

**Published:** 2016-06-21

**Authors:** Iiro P. Jääskeläinen, Juha Pajula, Jussi Tohka, Hsin-Ju Lee, Wen-Jui Kuo, Fa-Hsuan Lin

**Affiliations:** 1 Brain and Mind Laboratory, Department of Neuroscience and Biomedical Engineering, Aalto University School of Science, Espoo, Finland; 2 Department of Signal Processing, Tampere University of Technology, Tampere, Finland; 3 Department of Bioengineering and Aerospace Engineering, Universidad Carlos III de Madrid, Leganes, Spain; 4 Instituto de Investigación Sanitaria Gregorio Marañon, Madrid, Spain; 5 Institute of Neuroscience, National Yang Ming University, Taipei, Taiwan; 6 Institute of Biomedical Engineering, National Taiwan University, Taipei, Taiwan

## Abstract

Humor is crucial in human social interactions. To study the underlying neural processes, three comedy clips were shown twice to 20 volunteers during functional magnetic resonance imaging (fMRI). Inter-subject similarities in humor ratings, obtained immediately after fMRI, explained inter-subject correlation of hemodynamic activity in right frontal pole and in a number of other brain regions. General linear model analysis also indicated activity in right frontal pole, as well as in additional cortical areas and subcortically in striatum, explained by humorousness. The association of the right frontal pole with experienced humorousness is a novel finding, which might be related to humor unfolding over longer time scales in the movie clips. Specifically, frontal pole has been shown to exhibit longer temporal receptive windows than, e.g., sensory areas, which might have enabled processing of humor in the clips based on holding information and reinterpreting that in light of new information several (even tens of) seconds later. As another novel finding, medial and lateral prefrontal areas, frontal pole, posterior-inferior temporal areas, posterior parietal areas, posterior cingulate, striatal structures and amygdala showed reduced activity upon re-viewing of the clips, suggesting involvement in processing of humor related to novelty of the comedic events.

Humor is a multifaceted and highly interesting phenomenon that is an essential aspect of the social nature of human species. Understanding and sharing humor as well as laughing together plays an important role in social bonding and group formation^1^, possibly *via* an endogenous opioidergic mechanism^2^. Interpreting stressful situations as humorous further provides an important psychological coping mechanism for individuals and social groups, and allows presentation of conflicting information in a softer forms^3^. Humor has also been thought to act as a fitness indicator in sexual selection. Conversely, difficulties in understanding humor cause problems such as reducing one’s likeability by others and constraining one’s social life. Therefore, research into the neural basis of interpretation and experience of humor is clearly warranted.

Previous functional magnetic resonance imaging (fMRI) studies, by contrasting blood-oxygen level dependent signal elicited by relatively simple humorous *vs*. non-humorous stimuli such as cartoons^4^,^5^,^6^,^7^,^8^,^9^, drawings coupled with text providing humorous vs. non-humorous surprising interpretations^10^, short verbal passages^11^,^12^,^13^,^14^,^15^, and movie clips^4^,^16^,^17^, have disclosed a number of brain regions that participate in processing of humor. These have included inferior frontal gyrus^5^,^7^,^12^,^14^,^15^,^18^,^19^, middle frontal gyrus^5^,^8^,^20^, superior frontal gyrus^15^,^19^, middle temporal gyrus^4^,^7^,^20^, temporal pole^18^,^21^, left ventromedial prefrontal cortex^19^, supplementary motor area^18^, inferior parietal lobule^11^, temporal-occipital junction^21^, and subcortical structures, such as nucleus accumbens and amygdala^18^, as well as parahippocampal gyrus^19^.

Based on such findings, detailed models have been suggested about the specific roles of different brain regions in the process of understanding jokes, such as detection and resolution of incongruity^19^,^20^. For example, results of a recent meta-analysis suggested that detection and resolution of incongruity takes place in areas residing in the junction of temporal, parietal and occipital lobes, and responses related to rewarding effects of humor can be localized to midbrain dopaminergic structures^3^. One important aspect of the neural basis of humor that has been studied relatively little is which brain processes are relevant for processing of novelty of humorous events, *i.e.*, which neurocognitive processes underlie the phenomenon that jokes, whilst often still funny, are not as funny the second time one hears them.

Recent studies have shown that movies can successfully be used as naturalistic stimuli during fMRI^4^,^22^,^23^, thus providing an approach that is very well suited for studies on brain processing of real-life like humor in comedy movies. There are several complementary analysis approaches to achieve this. By aligning the brains of individual subjects and calculating correlations between the hemodynamic activity time courses for each voxel, one can inspect the degree of similarity in how the brains of individual subjects respond to common naturalistic stimuli such as movies. In previous studies that have utilized this inter-subject correlation (ISC) approach, significant ISC has been observed in both sensory cortical areas^23^,^24^,^25^,^26^,^27^,^28^,^29^ and hierarchically higher-order cortical areas such as the prefrontal cortex^24^,^25^,^26^,^27^,^28^,^29^ when subjects are watching movies during fMRI. The obvious strength of the ISC method is that one does not need to have any *a priori* models of the fMRI signal in response to complex movie stimulus in order to carry out the analysis, since the results are solely based on similarities in how the subjects’ brains react to the various aspects of the movie stimulus^26^. On the other hand, it was recently shown that ISC analysis detects relevant brain regions in highly structured experimental setups almost as sensitively as a model-based analysis^30^.

While an ISC analysis of fMRI data obtained during watching of a movie provides a map of brain areas that respond similarly across subjects, additional analyses are needed in order to determine which aspects of the movie stimulus and/or what experiences, such as shared emotional states elicited by the movies, explain the observed pattern of inter-subject similarity in hemodynamic signal. More specifically, self-reports of subjective experiences can be correlated with the ISC. As an example of this approach, fluctuations in self-reported emotional valence and arousal were successfully correlated with cortical ISC inspected over 20-sec sliding time window in a previous study^28^. A number of brain regions, including prefrontal ones, were disclosed wherein ISC co-varied with negative valence and elevated arousal^28^. Another data analysis approach that has been successfully used in movie studies and allows for pinpointing effects related to specific stimulus features in the movie and/or subjective states is general linear model (GLM) where specific stimulus feature time courses such as presence of human faces in the movie^31^ or emotions experienced by the subjects while watching movies^32^ have been used as predictors of hemodynamic activity.

To our knowledge, there have been only three previous studies where comedy-genre movie clips have been shown to healthy adult volunteers to map brain areas involved in processing of humor under naturalistic conditions. In the first study, episodes of “Seinfeld” and “the Simpsons” were shown to subjects during fMRI^4^. The laugh-track of the Seinfeld and a separately obtained laugh-track of the Simpsons episode were used as predictors in the analysis of the fMRI data. Analysis of time periods two seconds prior to onset of, and during, laughter on the laugh-track disclosed brain areas involved in detection and resolution of incongruity, including bilaterally posterior middle temporal gyrus, inferior frontal gyrus, insula, and amygdala. Hemodynamic activity during the laugh-track events was further seen unilaterally in the right cerebellum and in the left-hemisphere anterior temporal cortex, thalamus, lateral parietal cortex, and hippocampus^4^. In this early study, however, subjective ratings of experienced humorousness were not obtained from the subjects, but rather the overt reactions of separate studio (Seinfeld) and experimental (the Simpsons) audiences were used in the fMRI analysis. In another pioneering study, amusing film clips were shown to the subjects^16^ both during in-scanner rating of humorousness and passive watching of the clips with a subsequent separate experienced humorousness rating session. In the passive viewing condition, activations of right posterior insula, middle and superior temporal gyri bilaterally as well as cuneous were observed as a function of experienced humorousness. When the subjects rated their degree of experienced humorousness while they watched the movie clips during fMRI, these areas were also observed to be activated, and additionally there were significant prefrontal, anterior cingulate gyrus, thalamic, cerebellar, and striatal activations^16^.

In the third study, short 24-sec movie clips of stand-up-comedy were shown to volunteers during fMRI^33^. Individual ratings of humorousness were obtained from the subjects after each clip, and activations in certain brain areas additional to those observed in^4^ were documented^33^. These findings are in line with other studies wherein humoristic material other than movie clips was used as stimuli (*e.g.*, cartoons) and subjective ratings of humorousness were used as predictors in the fMRI data analysis. Looking across these studies, the additional areas active during experienced humorousness included dorsolateral prefrontal cortex^5^,^14^, fusiform gyrus^5^, cingulate gyrus^14^, supplementary motor area^33^, medial-ventral prefrontal cortex^12^, and subcortically putamen^5^,^14^,^33^, as well as nucleus accumbens^5^.

In the present fMRI study, we set forth to investigate which brain areas respond similarly when watching relatively long (~5 min) movie clips of comedy genre. The movie clips were taken from comedy-genre movies “The Circus” and “City Lights”, directed by Charles Chaplin and produced by Charles Chaplin Productions in 1928 and 1931, respectively. We hypothesized that, in addition to sensory cortical areas, we would observe significant ISC in brain regions implicated as humor-related areas in previous fMRI studies. In addition to this, we hypothesized that between-subject similarities in self-rated experienced humorousness explain similarities in hemodynamic activity in brain areas involved in generation of experienced humorousness when viewing the comedy-genre movie clips. Furthermore, we hypothesized that GLM analysis with experienced humor ratings as predictors discloses similar brain regions as the ISC analysis approach and that there are several brain regions that will show diminished humor-related hemodynamic responses when the subjects watch the comedy clips for the first time *vs*. when the clips are re-shown. Here we assume that novelty related to building up of expectations and subsequent violation of those expectations, which constitutes one of the core methods of joke-telling^34^, is reduced during re-viewing. To validate this assumption, we carried out a separate behavioral control experiment where subjects viewed the movie clips twice and rated their self-experienced humorousness on both views.

## Results

**Figure 1:**
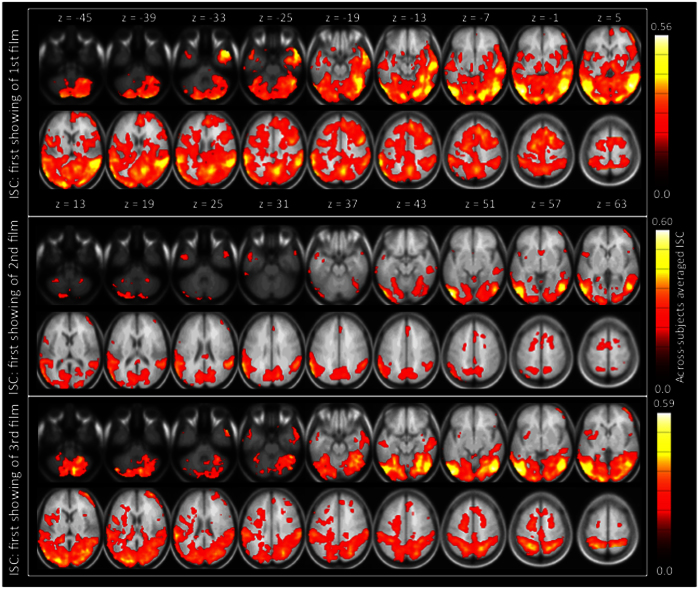
Group-level ISC during the first showing of the three comedy movie clips. As can be seen, ISC was observed in multiple cortical areas, and that the areas showing significant ISC were rather consistent across the three movie clips. Sensory cortical ISC suggest that the subjects visually sampled the movies in a similar fashion. Note, however, that it cannot be concluded based on this analysis which of the ISC effects were specifically associated with humor experienced by the subjects. (ISC was thresholded at the level Bonferroni P < 0.01, voxel-wise corrected over the whole brain.)

Consistent patterns of significant ISC were observed in both sensory and higher-order cortical areas over viewing of the three different movie clips. This is seen in Fig. 1 that displays maps of statistically significant ISC overlaid on top of axial slices of across-subjects averaged anatomical images. The ISC maps shown in this figure were calculated from hemodynamic activity recorded during the first viewing of the three movie clips. Voxel-wise spatial Pearson correlation coefficients between ISC maps over the whole brain were r = 0.82 (between first clip and second clip), r = 0.83 (between first clip and third clip), and r = 0.81 (between second clip and third clip), indicating high consistency in areas showing ISC across the clips (an overlap map and all other fMRI results can be browsed online at: http://neurovault.org/collections/VFXTWYOO/).

**Figure 2:**
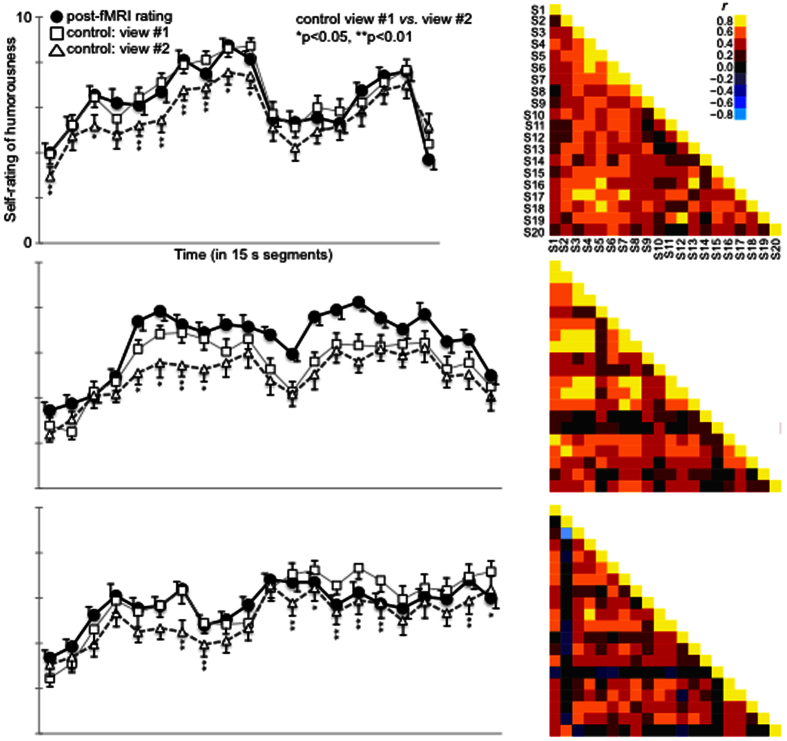
Self-reported humorousness of the three movie clips. LEFT) shown are self-reported humorousness values on a Likert scale 1–10 with zero denoting no experienced humorousness and 10 highest possible amount of experienced humorousness, obtained at steps of 15 seconds from the beginning to the end of each clip. Solid black lines depict the mean of self-ratings by subjects who immediately after the fMRI scans were re-shown the clips and rated the humorousness they recalled having experienced during the first viewing of the clips in the fMRI scanner. Solid gray and dotted black lines depict mean of self-ratings by subjects in the behavioral control experiment where the subjects rated their experienced humorousness during first and second viewing of the movie clips. Error bars show the standard errors of the mean (SEM). As can be seen, time-course of the across-subjects averaged experienced humorousness is rather similar between the two groups for each of the three movies. Further, it can be seen how intensity of experienced humorousness decreases going from the first to the second viewing of the movie clips in the control group (*p < 0.05, **p < 0.01). RIGHT) Shown are pair-wise inter-subject correlations of self-rated humorousness, as can be seen many of the subject pairs experienced humorousness rather similarly, but there were also subject pairs who experienced the humorousness of the clips quite differentially.

The self-reports of experienced humorousness (that were obtained once every 15 seconds during re-watching of each of the movie clips immediately after the fMRI session, as well as the ones obtained during first and repeated viewing of the same clips in the separate behavioral control experiment) are shown in Fig. 2. Overall, it can be seen that some parts of the clips were experienced as funnier than others. There was also some inter-subject variability in the degree of experienced humorousness as disclosed by the standard errors of the mean (SEM). Correlations between humorousness ratings for each pair of subjects are also shown in Fig. 2. These show that there were inter-individual differences in the experienced humorousness between some pairs of subjects but also high degree of similarity between some subjects. Kendall’s W, a statistical summary measure of inter-rater agreement, was for the three movie clips 0.52, 0.47, and 0.47 (p < 0.001 for each), implying significant overall agreement between the subjects in their ratings. Further, the fMRI and control group seemed to rate the humorousness highly similarly. Pearson correlations between the post-fMRI across-subjects averaged self-rating time course and that obtained during the first viewing of the same clip in the control experiment were r = 0.96 for the first clip, r = 0.92 for the second clip, and 0.91 for the third clip (p < 0.001 for each). In case of the first clip, there was significantly higher (p < 0.05) correlation between ratings given to first viewing in the fMRI group and the first viewing in the control group, than between ratings given to first viewing in the fMRI group and the second viewing in the control group. For the other two clips, the direction of the effect was the same, but the effects failed to reach significance. Further, while the same humorous events were identified during the second viewing of the clips in the behavioral control experiment, the self-reported intensity of experienced humorousness was significantly weaker during the second viewing of the clips as compared with the first viewing (p < 0.001, p < 0.05, and p < 0.01 when tested across all time points for the first, second, and third clip, respectively).

**Figure 3:**
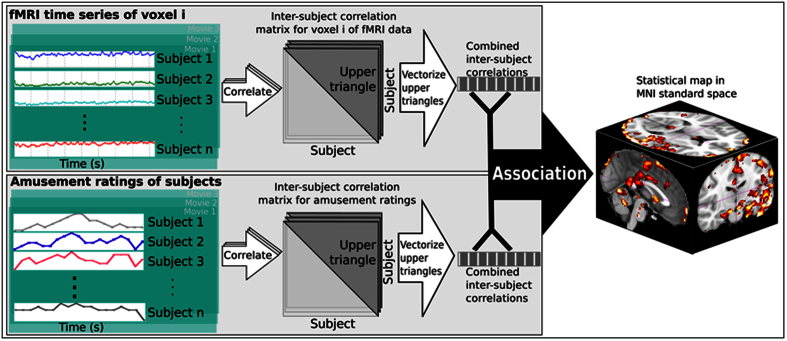
Description of the steps of the Mantel test with which the association between inter-subject similarity in experienced humorousness and inter-subject similarity in brain hemodynamic activity is measured. Subject-pair wise ISCs of brain hemodynamic time series (over the duration of the movie clip) are first calculated for each voxel. The resulting vector of ISCs is then correlated with vector of subject-pair wise ISC of humorousness self-ratings. This step is repeated for each voxel and overlaid on across-subjects averaged anatomical volumes to render three-dimensional statistical parametric maps.

**Figure 4:**
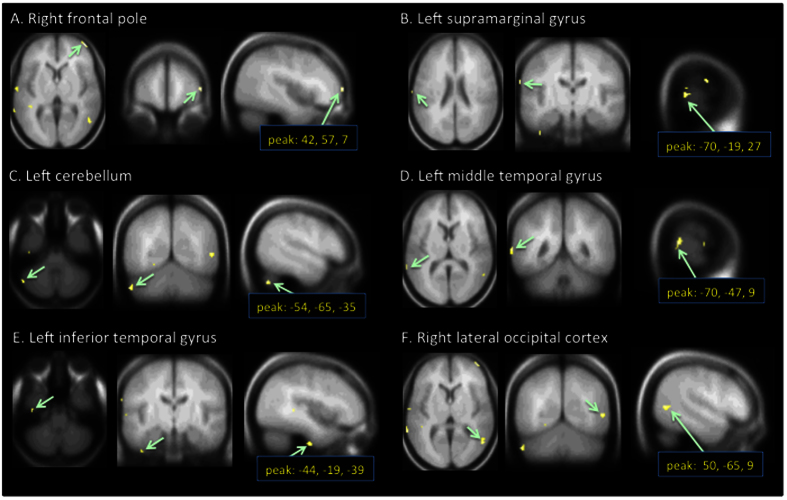
Results of the Mantel test showing the six brain regions wherein subject pair-wise ISC was found to be associated most significantly with across-subjects similarities in self-reported amount of humorousness elicited by the movies. Listing of all areas can be seen in Table 1. (Voxel-wise FDR corrected over the whole brain at q < 0.05; the peak coordinates are MNI coordinates).

The self-ratings of experienced humorousness were utilized in two distinct ways in the fMRI data analysis to disclose brain regions wherein hemodynamic signal is explained by experienced humorousness. First, a Mantel test was carried out where between-subject similarities in self-rated humorous experience were used to explain similarities in hemodynamic activity^35^,^36^ (see Fig. 3 for an illustration of the steps in this test). Six brain regions most significantly observed in this analysis are shown in Fig. 4 and the MNI coordinates of the significant peak voxels are listed in Table 1. There were several brain regions in which between-subjects similarity in self-rated humorousness explained ISC: the right hemisphere frontal pole, lateral occipital cortex, and middle temporal gyrus, in the left hemisphere supramarginal gyrus, lateral cerebellum, middle temporal gyrus, inferior temporal gyrus, angular gyrus, post-central gyrus, planum temporale, temporo-occipital fusiform cortex, lateral occipital cortex, lingual gyrus, and parietal operculum.

**Figure 5:**
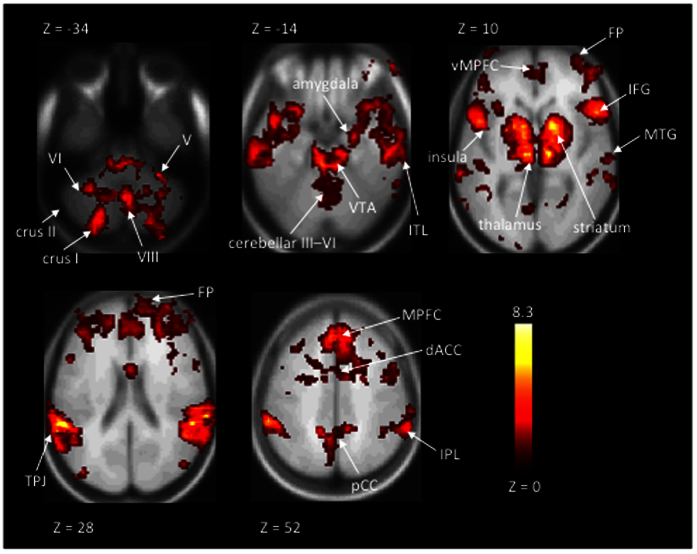
Statistical parametric maps of hemodynamic activity during first-time viewing of the movie clips as explained by self-rated humorousness in the GLM based analysis. As can be seen, experienced humorousness was associated with hemodynamic activity in a large number of anatomical structures, including cerebellar, subcortical, frontal, temporal, and parietal areas. Threshold at q < 0.05, voxel-wise FDR corrected across the whole brain. (Roman numerals refer to respective cerebellar areas; VTA = ventral tegmental area; ITL = inferior temporal lobe; vMPFC = ventromedial prefrontal cortex; MPFC = medial prefrontal cortex; FP = frontal pole; IFG = inferior frontal gyrus; MTG = middle temporal gyrus; TPJ = temporoparietal junction; dACC = dorsal anterior cingulate gyrus; pCC = posterior cingulate gyrus; IPL = inferior parietal lobule).

In addition, we used GLM to determine in which brain areas the self-ratings of humorousness explained brain hemodynamic activity during first viewing of the movie clips. Figure 5 shows that there were a number of areas activated during segments of self-experienced humorousness. There were significant bilateral activations in striatal structures that have been associated with processing of reward, in several prefrontal, temporal, and parietal areas, as well as cingulate cortex, and amygdala. MNI coordinates of the cluster peak voxels of significant activations in this analysis are listed in Table 2.

**Figure 6:**
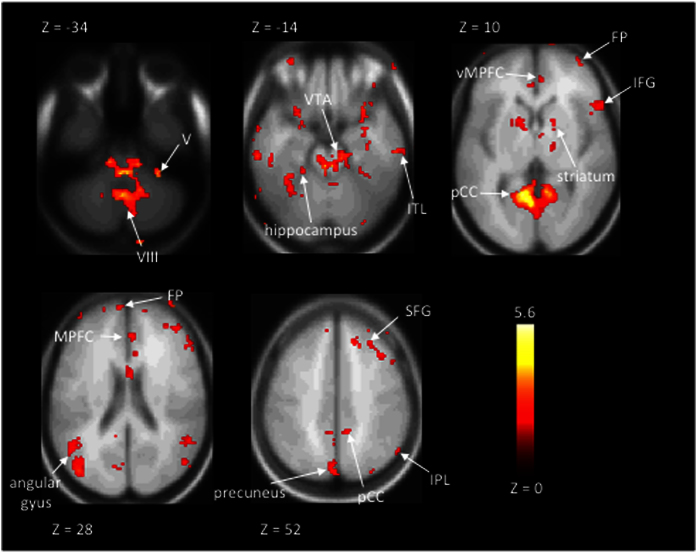
Statistical parametric maps of significantly higher hemodynamic activity during first-time viewing *vs*. second-time viewing of the movie clips as explained by self-rated humorousness in the GLM analysis. As can be seen, experienced humorousness was associated with hemodynamic activity in a large number of anatomical structures, including cerebellar, subcortical, frontal, temporal, and parietal areas. Threshold at q < 0.05, voxel-wise FDR corrected across the whole brain. (Roman numerals refer to respective cerebellar areas; VTA = ventral tegmental area; ITL = inferior temporal lobe; vMPFC = ventromedial prefrontal cortex; MPFC = medial prefrontal cortex; FP = frontal pole; IFG = inferior frontal gyrus; SFG = superior frontal gyrus; pCC = posterior cingulate gyrus; IPL = inferior parietal lobule).

Figure 6 shows the areas which exhibited in GLM analysis higher hemodynamic activity during the first viewing of the humorous movie clips than during the second viewing of the clips, *e.g.*, when the building-up of expectancies, and subsequent violation of the expectancies (in a funny way), had lost their novelty. As can be seen, there were significant bilateral activations in striatal structures, in several medial and lateral prefrontal cortical areas, inferior temporal, cerebellar, posterior cingulate, and parietal areas. MNI coordinates of the cluster peak voxels of significant activations in this analysis are listed in Table 3. We failed to see any significant activations in the opposite contrast (*i.e.*, testing for stronger humor-related activity during the second than during first viewing).

## Discussion

Previous fMRI studies have suggested several subcortical, parietal, temporal, and prefrontal brain regions as important for interpretation of jokes and the associated experienced humorousness^5^,^12^,^14^,^21^,^33^ (for a recent review, see^3^). In the present study, we examined which brain areas show across-subject replicable hemodynamic activity when healthy subjects are watching three ~5-minute movie clips taken from comedy films the “The Circus” and “City Lights”, both directed by Charles Chaplin and produced by Charles Chaplin Productions in 1928 and 1931, containing a variety of non-verbal humor. Further, to pinpoint areas related to processing of humor, we tested 1) in which brain areas the degree of subjectively experienced humor predicts the degree of ISC of hemodynamic activity, 2) in which brain areas the self-ratings of humorousness predict hemodynamic activity in a model based analysis, and 3) in which brain areas humor-related hemodynamic activity is decreased when going from the first-time viewing to the repeated viewing of the movie clips, *e.g.*, when the detection of incongruity and resolution of it had lost their novelty^34^. Here, it has to be noted as a cautionary remark that not all humor in the video clips involved detecting of incongruity and finding resolution of it, but also contained, e.g., jokes based on the building up and violation of expectations of the movie character that are fully expected by movie viewers.

In line with the findings of a number of previous studies^23^,^24^,^25^,^26^,^27^,^28^,^29^, we observed significant ISC in multiple sensory areas of the brain when the subjects watched the movies, indicating that the subjects were visually sampling each of the three movie clips in a highly similar manner (see Fig. 1). We also observed significant ISC in a number of brain regions that have been suggested to participate in processing of humorous stimuli in previous studies with simplified stimuli^5^,^6^,^7^,^8^,^9^,^11^,^12^,^13^,^14^,^15^ and movie stimuli in adults^4^,^16^,^33^ and children^17^. As can be seen in Fig. 1, significant ISC was consistently observed across the three different comedy movie clips that were shown in the present study. Naturally, the fact that we observed significant ISC in a replicable manner across the three comedy clips in a given brain region does not necessarily mean that the brain region is involved in processing of humor, since each of the comedy clips contain a wealth of stimulus features and information unrelated to the humorous content. Further, as we have shown previously, ISC calculated across the whole time series might miss brain areas showing similarity specifically during emotional states^28^. Given this, lack of ISC in a brain area does not necessarily implicate that the brain area is not involved in humor processing as there were a number of time windows in the clips with relatively low experienced humor (see Fig. 2).

In the present study, we attempted to pinpoint brain regions specifically involved in elicitation of subjectively experienced humor. As a cautionary remark, one has to keep in mind that since emotional processing is partly unconscious, self-reported values may represent limited aspect of experiencing humorousness. We first carried out a Mantel test^35^,^36^, where between-subject similarities in self-rated experienced humorousness were used to explain between-subject similarities in hemodynamic activity in the brain regions that showed significant ISC. This test revealed that hemodynamic activity in multiple left and right-hemisphere areas was associated with experienced humorousness (see Fig. 4; for a comprehensive list of areas disclosed by this analysis see Table 1). Of the observed areas, the association of the right frontal pole with experienced humorousness is a novel finding. Naturally, the sensitivity of the Mantel test between ISC and experienced humorousness is subject to similar shortcoming as the ISC calculated over the duration of the clips: there might be ISC in specific time windows of higher level of experienced humorousness that the analysis might miss.

To complement this analysis, we tested with GLM which brain regions are associated with experienced humorousness. The brain regions showing significant activations in this analysis are shown in Fig. 5 and listed on Table 2. As can be seen, the involvement of the frontal pole in humor processing was confirmed by the GLM analysis. Furthermore, an array of additional areas were observed to be associated with experienced humorousness, including extensive cerebellar, temporal, parietal, and prefrontal cortical areas, as well as striatal structures and amygdalas subcortically, thus supporting previous observations^5^,^12^,^14^,^33^. Observing these additional areas suggests that the GLM analysis was more sensitive than the Mantel test. Of these, the activation of striatal structures have been linked to rewarding effects of experienced humor in previous studies and areas in the junction of temporal and parietal lobes have been linked with the resolution of incongruence in humor^3^. However, it should be noted that the Mantel test and GLM analysis test different (although related) hypotheses and straightforward comparison of the results of these analyses is not necessarily possible.

The finding of significant ISC and hemodynamic activity associated with experienced humorousness in frontal pole is a new one and might, tentatively, be related to processing of humor unfolding over longer time scales in the relatively long comedy clip stimuli. This is theoretical speculation that should be viewed with caution, however, it is based on recent findings showing that especially anterior prefrontal regions integrate incoming higher-order information over longer temporal receptive windows than for example primary and secondary sensory areas^37^,^38^,^39^. In the case of experienced humor, it is often the case that understanding a joke is based on keeping in mind preceding information and reinterpreting that information in the light of new incoming information several seconds or even a few tens of seconds later. Temporal receptive windows longer than those in sensory areas are needed to successfully carry out such operations^39^.

Using GLM we further tested in which brain regions hemodynamic activity associated with experienced humorousness was diminished going from the first to the repeated viewing of the comedy clips. Here, we assumed that such effects take place due to decreasing novelty, as novelty is important for humorousness (and indeed the results of our behavioral control experiment shown in Fig. 2 confirmed that experienced humorousness was diminished for the second viewing of the clips). The results of this analysis are shown in Fig. 6 and Table 3. As can be seen, there were significant bilateral activations in striatal structures, in several medial and lateral prefrontal cortical areas including frontal pole, right anterior insula, amygdala bilaterally, inferior temporal, cerebellar, posterior cingulate, and a number of parietal areas. It is possible that these areas are especially important for solving the humor-related incongruence and the rewarding effects of comedic events when they are seen for the first time. Notably, the fact that frontal pole was observed also in this analysis add further support for the notion that frontal pole is associated with experienced humorousness.

Across the three previous studies wherein comedy-genre movie clips have been used as naturalistic stimuli in healthy adult volunteers^4^,^16^,^33^, humor-related activity was observed in or in the vicinity of most of the brain regions that were observed in the present study. Some of the areas observed in the present study were not, however, seen in these three previous studies. Specifically, the hemodynamic activity associated with self-ratings of experienced humor in frontal poles (see Figs 4, 5, 6) does not seem to match any of the activation foci documented in the previous studies that used comedy-genre movie clips^4^,^16^,^33^. While it is difficult to say with certainty what might have caused these discrepancies in findings, it is possible that they stem from differences in experimental design. In one of the previous studies the laugh track obtained from separate groups of individuals was used as the humorousness predictor in a model-based analysis^4^, whereas in our study, self-reported subjective experiences of humorousness of the scanned subjects were utilized in the ISC and GLM based analysis approaches. As suggested by relatively large inter-subject variability in experienced humorousness of the movie clips in our study involving some of the subject pairs (see Fig. 2), humor might be one of the more subjective experiences and thus using an independently derived model of humorousness in fMRI data analysis might result in a failure to see some effects.

Furthermore, in the other one of the previous studies where movie clips were used as stimuli^33^, the duration of the clips was 24 seconds which is considerably shorter than the approximately five-minute clip duration that were used in the present study. Thus, the shorter 24-sec clips might have lacked development of humorous events over longer timescales that have been observed to be processed in anterior prefrontal areas, including the frontal poles^27^,^37^. As a further experimental design difference that might explain differences in findings, the subjects rated the humorousness of each of the 24-sec clips immediately after having seen them during the fMRI scanning in the previous study^33^, whereas in the present study the clips were rated only after the fMRI session without any advance warning, thus allowing for more natural free viewing conditions. In a previous study where passive viewing and viewing during rating of humorousness were directly contrasted, both augmentation of activity and activation of additional areas was observed during in-scanner rating^16^.

While it is tempting to speculate which of the observed brain areas participated in which aspect of humor processing, such as resolution of incongruence, our experimental setup was limited in the sense that it does not make it possible to answer such questions directly. According to a recent meta-analysis, temporal-parietal junction (TPJ) is an important area that deals with resolution of incongruence that often is an important constituent of humorous events^3^. The fact that we observed activity related to humor novelty in or in the vicinity of TPJ (see Fig. 6) tentatively suggests that the effects we observed therein were due to resolution of humor-related incongruence in the course of watching the movie clips. The left-hemisphere inferior temporal regions might have, in turn, been due to higher-order visual-semantic processing, though these areas have been less saliently associated with specific aspects of humor processing in previous studies^3^. As a further cautionary remark, one has to keep in mind that we cannot completely rule out that some of the observed humor-novelty effects could have been due to non-specific effects of stimulus novelty. There are previous studies that have presented naturalistic stimuli with inter-stimulus intervals comparable to those in the present study between first and repeated presentation of a movie clip (*i.e.*, several minutes; it is in fact important to realize that typical repetition-suppression effects are unlikely to explain the present findings as such mostly occur with inter-stimulus intervals of ~seconds). In such studies, novelty effects (unrelated to experiencing humor) have been observed in superior-lateral temporal cortices and insula bilaterally as well as in left inferior frontal gyrus^40^. Given the fact that there were considerable inter-individual differences in which scenes were experienced humorously in the present study, the fact that the hemodynamic effects were significantly associated with this variability in the analyses that were employed, and the fact that such associations were diminished upon repeated viewing of the clips as experienced humorousness diminished, suggests genuine involvement of the areas in experiencing humorousness and the diminution of that as the novelty of the humor washed away. This especially applies to our novel observation of the involvement of frontal pole in experienced humorousness during watching of movie clips, given that the frontal pole was consistently observed across all these analyses.

In conclusion, our study expands research on the neural basis of humor in several ways. First, as a novel finding, we observed that the frontal pole is synchronized across subjects and exhibits increased hemodynamic activity in association with self-experienced humorousness. Naturally, caution needs to be exercised when speculating on the possible underlying mechanisms, but it is possible that these anterior frontal lobe areas are involved in processing humor that occurs over longer timescales in the relatively long movie clips used in the present study, given that previous studies have shown that anterior prefrontal regions process stimulus events over longer timescales than sensory cortical areas. As the second novel finding, we showed that multiple areas in prefrontal, inferior temporal, and parietal cortices as well as subcortically striatal structures that are associated with humor processing were diminished in activity upon re-viewing of the movie clips when novelty of the humorous events had been washed away.

## Methods

### Subjects

20 healthy volunteers (age range: 20–25, 7 females, normal or corrected to normal vision) participated in the main experiment that consisted of fMRI scanning session and behavioral self-ratings. Two subjects were excluded from fMRI data analysis due to technical problems with their fMRI data. A separate group of 18 healthy volunteers (age range: 20–29, 9 females, normal or corrected to normal vision) participated in a behavioral control experiment (for details of the main and control experiments, see below). A voluntary informed consent was obtained from each of the subjects prior to participation, both experiments were run in accordance with the guidelines of the Helsinki Declaration, and ethics approval was obtained from the Ethics Committee of the National Yang Ming University prior to commencing the research.

### Stimuli and MRI acquisition

In the main experiment, the subjects were presented with three short silent movie clips twice, in an order that was randomized across subjects, during 3T (Tim Trio, Siemens, Erlangen, Germany) fMRI with a 32-channel head coil. (Echo-planar imaging (EPI); TR/TE = 2000/30 ms, field-of-view = 220 × 220 mm, matrix = 64 × 64, in-plane 3.4 × 3.4 mm, slice thickness = 3.4 mm, flip angle = 90°). Thirty-three axial slices were acquired to cover the whole brain. The movie clips were taken from comedy-genre movies “The Circus” and “City Lights”, directed by Charles Chaplin and produced by Charles Chaplin Productions in 1928 and 1931, respectively. The two clips from “The Circus” (https://www.youtube.com/watch?v=viWyAxWuQT0) were taken from 6’30” and 58’32” of the movie for 4’33” and 5’08”, respectively. One clip from “City Light” (https://www.youtube.com/watch?v=0io7GfiHmX4) was taken from 1:03’01” of the movie for 5’17”. The clips were displayed in fMRI with a projector (PT-D5500U, Panasoic, Osaka, Japan) onto a semi-translucent screen fitted to the bore of the MRI scanner. Participants watched movie clips via a reflecting mirror mounted on the head coil array. We obtained 145, 166, and 162 fMRI volumes during each movie clip. There were pauses of two minutes between the runs in order to wash out the effects of the preceding clip. Prior to the fMRI, a high-resolution *T*_1_-weighted anatomical (MPRAGE sequence, TR/TI/TE/flip = 2530 ms/1100 ms/3.03 ms/7^o^, partition thickness = 1 mm, matrix = 256 × 256, 192 partitions, FOV = 22.4 cm × 22.4 cm) scans were obtained from each subject.

### Self-reported humor ratings

In the main experiment, the subjects were re-shown, immediately after the fMRI session, and without advance warning, the three silent comedy clips on a PC screen and were asked to recall and rate, on a Likert scale running from 1 to 10, once every 15 seconds, the degree of humorousness that they had experienced due to the humorous events depicted in the movies. During rating, a 15-s clip segment was first shown to the participant. Then the rating program waited for the participant’s rating on a 10-point Likert scale. After completing the rating, the rating program automatically started the showing of the next 15-s clip segment. This procedure was repeated until the last 15-s clip segment was shown and the participant’s rating was collected. We used E-Prime 2.0 (version 2.0.8.90, Psychology Software Tools Inc., Sharpsburg, PA, USA) to control the presentation of movie clips and the acquisition of behavioral responses for both fMRI and control experiments. Note that the participants were specifically instructed to recall the humorousness they had experienced during the first viewing of the movie clips that took place during fMRI scanning. This method is analogous to what we have used in our previous fMRI studies on cerebral basis of experienced emotions (Jääskeläinen *et al*.^24^; Nummenmaa *et al*.^28^). A rating of one corresponded to lack of experienced humor and a rating of ten corresponded to high degree of experienced humor. In the control experiment, the separate set of 18 subjects viewed the three movie clips twice. They were instructed to rate the degree of humorousness they experienced while viewing the three clips for the first time, with the self-rating taking place after each 15-sec segment, followed by viewing and rating of the three clips for the second time. The order in which the clips were shown was identical to that in the fMRI experiment. The control experiment was specifically designed to test to what extent the degree of experienced humorousness is altered from the first to second viewing of the humorous clips.

### Data analysis

EPI data were first pre-processed with FSL (http://www.fmrib.ox.ac.uk/fsl/, version 4.0) by applying motion correction, slice-timing correction, and spatial smoothing (3D isotropic Gaussian kernel with the full-width-half-maximum = 6 mm). Subsequently, individual-subject EPI data were spatially registered to individual anatomical data using the 12-parameter affine transformation as implemented in FSL. Finally, across-subject morphing was accomplished by registering anatomical images to MNI space at 2 mm isotropic resolution.

ISC was calculated separately for fMRI data obtained during each movie clip for the first and second viewing using our previously published toolbox (ISC-Toolbox, version 2.1)^27^,^41^. Before calculation of ISC, we first removed the time-invariant constant value and the linear trend in each EPI time series using General Linear Model. The ISC for each movie was computed as the voxel-wise mean correlation of the BOLD time series across all pairs of subjects as detailed in^41^. The statistical inference was accomplished by a fully nonparametric voxel-wise resampling test^27^ and a formal description of the test can be found in^42^. The resampling test constructs the null-distribution of the ISC values by circularly shifting the time series of each subject by a random amount and thus breaking the simultaneous timing between the time series of different subjects while retaining other characteristics of the time series. The full resampling distribution was approximated with 100 000 000 realizations, sampling randomly across the brain voxels for each realization. The resulting ISC maps were thresholded at Bonferroni-corrected level of p = 0.01 and overlaid on the MNI space. After this, we tested in which brain areas inter-subject similarity in self-ratings of experienced humorousness was associated with inter-subject similarity of brain activity. To achieve this, we calculated inter-subject correlation of the self-reported experienced humorousness for each subject pair and used a Mantel test to determine the extent that these pair-wise correlations explained the pair-wise ISC. A Mantel test makes inferences about association between two distance (or similarity) matrices^43^. Here, we modified the Mantel test to combine data across the three different movie-viewing sessions and for allowing multiple comparisons correction with a reasonable computational cost. The basic logic of this analysis approach is illustrated in Fig. 3.

Specifically, the modified Mantel test was carried out as follows: for each voxel *v*, **I**_j_(*v*), *j* = 1,2,3, is the Fisher’s z-transformed N x N intersubject correlation matrix of hemodynamic responses for the movie *j* and the first viewing session. Let **C**_j_ be the Fisher’s z-transformed N x N correlation matrix of experienced humorousness ratings for the movie *j* across N subjects. N = 18 denotes the number of subjects. We de-meaned the matrices **I**_j_(*v*) so that their average over the brain voxels was a zero matrix. The de-meaned matrices are **F**_j_(*v*) = 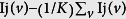 and K is the number of brain voxels. The purpose of the de-meaning was to prevent the unwanted influence of the average BOLD-ISC between two subjects to the Mantel statistics that biases the Mantel test statistic if the average BOLD-ISC correlations were (by chance) correlated with humorousness rating correlations. Let ut(**A**) denote the operation of extracting the upper triangular part of a matrix **A** and vectorizing it. Applied to an 18 × 18 correlation matrix **C**, this operation produces a 153-element vector of all the distinct correlation values in the matrix **C**. For a voxel *v*, the test statistic of the modified Mantel test *M*(*v*) measures the association between the vectors **f**(*v*) = 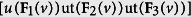 and ***a*** = 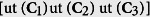 and we use the notation *M*(*v*) = Assoc(**f**(*v*),**a**). To measure the association, we applied Pearson correlation to test the relation between the humor ratings and the ISC during the first viewing,


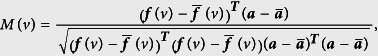


where 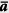 (resp. 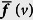) denotes the average of the elements of the vector *a* (***f***(***v***))

To form a distribution for the test statistic under the null-hypothesis that the mechanism that generates pair-wise humorousness-rating correlations is independent from the mechanism that generates pair-wise ISCs of hemodynamic activity, rows and columns of each of the three self-report correlation matrices were first randomly permuted as in the Mantel test^43^. Thereafter, the three permuted matrices were randomly re-ordered and the test-statistic distribution under the null-hypothesis was formed by repeating the process twenty million times by randomly sampling a different voxel each time. Only those voxels that exhibited small ISC values for all movies (*i.e.*, voxels whose ISC values were smaller than the FDR corrected ISC threshold with q = 0.05 for all movies) were sampled when calculating the null-distribution. Note that the FDR corrected threshold with q = 0.05 is a very liberal ISC threshold and particularly it is more liberal than Bonferroni corrected threshold with *p* = 0.01 used in Fig. 2. In more detail: Let the operation perm (*N*) return a random permutation of the integers 1, …, *N*. To approximate the null-distribution P_M_ for M, the following extension of the standard Mantel-matrix re-sampling scheme is utilized:

Repeat following steps 20 million times:

1. Sample a voxel u that does not show significant ISC for any movie
2. Permute the rows and columns of **C**_j_ with the same permutation resulting in **C**_jr_, *i.e.*, compute:for *j* from 1 to 3**p** ← perm(N)**C**_jr_ ← **C**_j_[**p**,**p**] // Rows and columns of **C**_j_ permuted with the same permutationend
3. Permute **C**_1r_, **C**_2r_, **C**_3r_ resulting in **C**_1rr_, **C**_2rr_, **C**_3rr_, i.e., compute**p** ← perm (3)**C**_1rr_ ← **C**_**p**[1]r_**C**_2rr_ ← **C**_**p**[2]r_**C**_3rr_ ← **C**_**p**[3]r_
4. Compute Assoc ([ut (**F**_1_(u)) ut **F**_2_(u)) ut (**F**_3_(u))], [ut (**C**_1rr_) ut (**C**_2rr_) ut (**C**_3rr_)]) and add it to P_M_

The reason for restricting the set of voxels sampled to form the null distribution is that the variance of Mantel statistic increases with the increase of the average ISC. Thus, the idea is to reduce the effect of the high ISC voxels (located mainly in visual cortex) to the null distribution (sampling also these voxels widens the null distribution) and avoid a test that would be conservatively biased version of the already conservative Mantel test^44^. Naturally, this choice is reflected in the interpretation of null-hypothesis and obviously prevents using extreme statistics-based permutation procedures for multiple comparison correction. A better alternative would have been to compute a separate null-distribution for each voxel, but the high computational cost prohibits this.

To correct the resulting *p*-values for multiple comparisons, we applied the voxel-wise FDR correction over the whole brain at the threshold q = 0.05. We present the results as a Z-statistic map obtained by transforming an uncorrected p-value map into the corresponding Z-statistic map using the p-to-Z transform. Obviously, the p-value threshold (p = 0.00004 corresponding to the FDR threshold q = 0.05) applied to the map was transformed in the same way.

In addition to the model-free ISC analysis, we tested using GLM based analysis in which brain areas hemodynamic activity was associated with experienced humorousness during the first viewing of the three movie clips. Three level mixed effects statistical analyses were performed within FSL’s FEAT tool (version: 6.00)^45^. At the first (single subject and movie) level, we entered experienced humorousness ratings of each individual subject (along with their first temporal derivatives) as predictors in a GLM after convolution with the standard hemodynamic response waveform (a double gamma-function). Higher level group analyses, first across the subjects and then across the three movies, were performed using FSL’s FLAME (with the 1 + 2 option and the outlier de-weighting) using a mixed effects model for both the second and the third level analyses^46^. To correct for multiple comparisons, FDR correction was applied at q = 0.05 (voxel-wise across the whole brain). After this, we tested using GLM in which brain areas hemodynamic activity associated with experienced humorousness was reduced (or increased) going from the first-time viewing to the repeated viewing of the movie clips. We specifically assumed that brain hemodynamic activity associated with humorousness decreases when the building-up of expectancies, and subsequent violation of the expectancies (that are one important constituent of humor), lose their novelty. We entered experienced humorousness ratings of each individual subject as predictors (along with their first temporal derivatives) in a GLM after convolution with the standard hemodynamic response waveform, and contrasted first viewing against the second viewing of the clips. The group level statistical map were constructed as described above using a mixed effects model and FDR correction was applied at q = 0.05.

## Additional Information

**How to cite this article**: Jääskeläinen, I. P. *et al*. Brain hemodynamic activity during viewing and re-viewing of comedy movies explained by experienced humor. *Sci. Rep.* **6**, 27741; doi: 10.1038/srep27741 (2016).

## Figures and Tables

**Table 1:** Peak loci of ISC explained by self-rated humorousness in the Mantel test (listing limited to one peak per anatomical structure).

Brain region	*x*	MNI *y*	*z*	Peak Z-score
Frontal pole (R)	42	57	7	4.7
Supramarginal gyrus (L)	−70	−19	27	4.6
Cerebellum (L)	−54	−65	−35	4.5
Middle temporal gyrus (L)	−70	−47	9	4.4
Inferior temporal gyrus (L)	−44	−19	−39	4.4
Lateral occipital cortex (R)	50	−65	9	4.4
Angular gyrus (L)	−68	−45	17	4.4
Post-central gyrus (L)	−60	−19	27	4.2
Planum temporale (L)	−68	−13	9	4.2
Temporo-occipital fusiform (L)	−28	−41	−13	4.2
Lateral occipital cortex (L)	−40	−69	−1	4.2
Middle temporal gyrus (R)	54	−59	11	4.1
Lingual gyrus (L)	−26	−63	−5	4.1
Parietal Operculum (L)	−50	−31	27	4.0

**Table 2:** Peak loci of hemodynamic activity explained by self-rated humorousness in the GLM analysis during first-time viewing of the movie clips (listing limited to one peak per anatomical structure).

Brain region	*x*	MNI *y*	*z*	Peak Z-score
Supramarginal gyrus (L)	−60	−37	35	8.3
Precentral gyrus (R)	16	−33	41	5.3
Posterior cingulate gyrus	−12	−25	35	5.2
Frontal pole (R)	32	65	-15	4.8
Superior lateral occipital (L)	−40	−81	25	4.7
Superior lateral occipital (R)	48	−73	25	4.0
Inferior temporal gyrus (R)	54	−13	−35	3.3
Cerebellum (L)	−52	−67	−37	3.1
Temporal fusiform cortex (L)	−42	−23	−35	3.1
Precentral gyrus (L)	−40	−13	47	3.1
Frontal pole (L)	−20	69	−9	2.9
Superior parietal (R)	10	−65	63	2.9
Inferior temporal gyrus (L)	−52	−43	−23	2.8
Middle temporal gyrus (R)	74	−1	−15	2.7
Planum temporale (L)	−42	−39	15	2.7
Parahippocampal gyrus (R)	22	−11	−35	2.6
Cerebellum (R)	6	−91	−41	2.5
Dorsal anterior cingulate	2	−7	45	2.5
Lateral-inferior occipital (L)	−52	−75	−13	2.5
Middle temporal gyrus (L)	−72	−11	−19	2.5
Temporal pole (L)	−48	27	−21	2.5
Orbitofrontal cortex (L)	−46	31	−21	2.4
Rostral anterior cingulate	4	27	5	2.3

**Table 3:** Peak loci of significantly higher hemodynamic activity explained by self-rated humorousness in the GLM analysis during first-time viewing vs. second-time viewing of the movie clips (listing limited to one peak per anatomical structure).

Brain region	*x*	MNI *y*	*z*	Peak Z-score
Precuneous	14	−55	17	5.6
Brainstem	−2	−35	−21	5.4
Insula (L)	−30	23	−5	4.7
Posterior cingulate gyrus	−6	−45	39	4.7
Middle temporal gyrus (L)	−70	−17	−11	4.4
Cerebellum (R)	26	−97	−35	4.3
Frontal pole (L)	−28	39	39	4.3
Middle temporal gyrus (R)	62	−17	−15	4.2
Superior lateral occipital (L)	−42	−73	23	4.2
Temporal pole (L)	−42	21	−23	4.1
Paracingulate gyrus	4	39	37	4.0
Middle frontal gyrus (R)	44	35	39	4.0
Posterior parietal cortex (L)	−28	−81	37	4.0
Inferior lateral occipital (L)	−36	−79	−25	3.9
Frontal Pole (R)	28	43	45	3.9
Temporal-occipital junction (R)	70	−47	−7	3.6
Posterior parietal cortex (R)	34	−75	43	3.6
Superior frontal gyrus (R)	14	33	49	3.6
Inferior temporal gyrus (R)	60	−25	−33	3.5
Cerebellum (L)	−22	−89	−29	3.4
Occipital fusiform (R)	30	−79	−15	3.4
Orbitofrontal cortex (L)	−28	21	−21	3.3
Inferior frontal gyrus, pars triangularis (R)	54	31	9	3.3
Temporal fusiform cortex (L)	−34	−15	−33	3.2
Temporal pole (R)	54	15	−31	3.2
Occipital fusiform (L)	−8	−89	−21	3.2
Putamen (R)	30	5	3	3.2
Inferior frontal gyrus, pars opercularis (R)	58	21	21	3.2
Thalamus (L)	−2	−15	13	3.2
Hippocampus (L)	−32	−19	−15	3.1
Thalamus (R)	14	−15	7	3.1
Inferior frontal gyrus, pars triangularis (L)	−52	25	13	3.1
Anterior cingulate gyrus	−8	33	17	3.1
Cuneus	−4	−85	29	3.1
Temporoparietal junction (L)	−60	−67	29	3.1
Inferior lateral occipital (R)	30	−87	−29	3.0
Middle frontal gyrus (L)	−40	33	41	3.0
Temporal fusiform cortex (R)	34	−33	−23	2.9
Inferior temporal gyrus (L)	−56	−35	−15	2.9
Parahippocampal gyrus (L)	−26	−29	−13	2.9
Ventral striatum (L)	−8	−3	−3	2.9
Angular gyrus (L)	−30	−55	35	2.9
Supramarginal gyrus (R)	42	−33	35	2.9
Subcallosal cortex	4	29	−7	2.8
